# Nurses’ perceptions of mental health care for patients with malignant wounds

**DOI:** 10.1590/0034-7167-2025-0116

**Published:** 2026-06-12

**Authors:** Daniele Cristina Cordeiro Ferreira da Silva, Aline Mayara Cardoso Baía, Danielle Etienne de Oliveira Bezerra Lima, Dione Seabra de Carvalho, Mary Elizabeth de Santana, Marcos José Risuenho Brito Silva

**Affiliations:** I Faculdade Cosmopolita. Belém, Pará, Brazil; II Universidade do Estado do Pará. Belém, Pará, Brazil

**Keywords:** Oncology Nursing, Mental Health, Neoplasms, Wounds and Injuries, Palliative Care., Enfermería Oncológica, Salud Mental, Neoplasias, Heridas y Lesiones, Cuidados Paliativos.

## Abstract

**Objectives:**

to analyze nurses’ perceptions of mental health care for patients with malignant wounds.

**Methods:**

qualitative descriptive study; virtual interviews were conducted with 20 oncology nurses via Google Meet. Data were analyzed using qualitative content analysis (Bardin) and IRaMuTeQ (Descending Hierarchical Classification and similarity analysis).

**Results:**

five thematic classes emerged: 1) The relationship between wound dressing and mental health; 2) Malignant wounds and their impact on patients’ psychosocial health; 3) User embracement and active listening as strategies for mental health care; 4) The multidisciplinary approach and its weaknesses in mental health care; and 5) The relationship between age and coping with cancer and its complications.

**Final Considerations:**

according to nurses, mental health care encompasses wound dressing, symptom control, and factors that aggravate symptoms such as age and socioeconomic context. The importance of the multidisciplinary team in this care and its fragilities were highlighted.

## RESUMEN

**Objetivos:**

analizar la percepción de profesionales de Enfermería sobre la atención a la salud mental en pacientes con herida neoplásica.

**Métodos:**

estudio descriptivo cualitativo; 20 profesionales de Enfermería en oncología fueron entrevistados virtualmente por Google Meet. Los datos se analizaron mediante análisis de contenido (Bardin) e IRaMuTeQ (Clasificación Jerárquica Descendente y análisis de similitud).

**Resultados:**

emergieron cinco clases temáticas: Relación entre el apósito y la salud mental; Impacto de la herida neoplásica en la salud psicosocial; Acogida y escucha activa como estrategias de atención a la salud mental; Abordaje multidisciplinar y sus fragilidades de atención a la salud mental; y Relación entre edad y afrontamiento del cáncer y sus complicaciones.

**Consideraciones Finales:**

para los profesionales, la atención a la salud mental abarca manejo del apósito, control de la sintomatología y factores agravantes como edad y contexto socioeconómico. Se destacaron la importancia del equipo multidisciplinar y las fragilidades de su actuación.

## INTRODUCTION

Among cancer-related complications are malignant wounds, which differ from wounds of other etiologies because of their specific features. As tumors grow and invade healthy tissues, blood vessels become compromised, which leads to impaired oxygenation, hypoxia, and necrosis of the affected tissue. This process favors bacterial colonization, resulting in increased exudate and malodor^(1)^.

From the moment cancer is diagnosed, individuals’ mental health is markedly affected. For example, in a sample of 100 patients, 27% had depressive symptoms, 24% showed indicators of anxiety, and 13% had indicators of both conditions. Thus, symptoms of depression and anxiety are more prevalent among patients with cancer than in the general population because they live with pain, discomfort, uncertainty, and, often, physical disfigurement caused by lesions^(2)^.

Malignant wounds affect approximately 5%-10% of patients at some point during the disease course^(3)^. They directly affect patients’ mental state; distress is triggered by characteristic and stigmatizing symptoms-such as malodor, pruritus, and disfigurement-which cause profound discomfort and suffering. In this situation, appropriate care for symptom management is essential^(1)^.

People with malignant wounds require specialized care, given the wound’s complexity and specific features and the harm to their well-being. A multidisciplinary team should provide care; nursing care is essential in the wound-care process, which often becomes a complex task because of knowledge gaps regarding this type of wound^(4)^.

Despite numerous challenges, nurses play a central role in comprehensive care for these patients. The nursing team’s continuous presence fosters closer contact and understanding of patients’ needs. In addition to the steps inherent in the nursing process, nurses can use active listening as a tool to support patients’ biopsychosocial needs^(5)^.

We identified the scientific literature on this topic through a PubMed/MEDLINE search using the descriptors “Palliative Care”, “Injury”, “Wound”, “Neoplasms”, “Cancer”, “Mental Health”, and “Nursing”, combined with the Boolean operators AND and OR, with no time restriction. The search returned eight publications. After screening titles and abstracts, only one study examined the decline in quality of life and mental health among patients with malignant wounds, and it approached the topic from patients’ perspectives^(6)^. This finding underscores the need for studies addressing nursing care specifically aimed at the mental health of patients with malignant wounds.

Therefore, analyzing nurses’ perceptions is essential to generate evidence supporting better care for patients with malignant wounds.

## OBJECTIVES

To analyze nurses’ perceptions of mental health care for patients with malignant wounds.

## METHODS

### Ethical aspects

The study complied with Brazil’s National Health Council (CNS) Resolution 510/2016 (dated April 7, 2016)^(7)^ and the ethical principles outlined in CNS Resolution 466/2012 (dated December 12, 2012)^(8)^. The study protocol was approved by a Research Ethics Committee (REC). The Informed Consent Form (ICF) and the Audio Recording Authorization Form were completed and electronically signed by all participants.

### Study design

We performed a qualitative descriptive study. Descriptive studies characterize phenomena within a group or population, whereas qualitative approaches focus on understanding subjective meanings. This design assumes that human knowledge is best understood through experiences narrated by those who live them^(9,10)^. Across all phases, we adhered to the Consolidated criteria for reporting qualitative research (COREQ)^(11)^.

### Study setting

The study was conducted virtually using the free version of Google Meet, which facilitated access, broadened the geographic reach of professionals, and increased comfort, as participants joined from home at convenient times. No institutional affiliation was required for participation.

### Data source

Participants were contacted via WhatsApp, through which they received an invitation to take part in the study. During the initial contact, the team presented the project, explained the study’s objectives and rationale, and detailed the methodology, helping to build rapport between professionals and researchers.

Snowball sampling was used to compose the sample^(10)^, allowing access to additional contacts through referrals and recommendations. We included oncology nurses and oncology residents who were currently or had previously been involved in caring for patients with malignant wounds for more than three months. Nurses who were on vacation or medical leave during data collection were excluded. Of the 56 invitees, 32 declined to participate, and 4 withdrew before the interview, citing lack of availability.

The sample was defined by data saturation-that is, data collection ended when interviews no longer yielded new, relevant information^(12)^. Thus, the sample comprised 20 nurses.

### Data collection and organization

Data were collected in October and November 2024 through individual virtual interviews conducted by two undergraduate nursing students. Each interview lasted about 20 minutes and was audio-recorded for subsequent transcription. All data were stored in the cloud and will be retained for five years, after which they will be deleted. Participant anonymity was ensured by using the letter “N” (for “nurse”) followed by an Arabic numeral (N1, N2, N3…).

A semi-structured interview guide with 13 questions covered training and professional practice, experience in public or private services, and age range. Additional questions addressed psychological distress observed in patients and possible precipitating factors, strategies used to mitigate such harm, and challenges related to working within the multidisciplinary team, among others, allowing participants to freely elaborate on their perceptions and experiences.

### Data analysis

Transcription was performed using the voice-typing feature of the online Google Docs word processor, producing verbatim transcripts. Next, all data were collated, highlighting key points related to the topic. The interviews were analyzed and categorized using qualitative content analysis as proposed by Laurence Bardin, which comprises three stages: pre-analysis; exploration of the material; and data processing and interpretation^(13)^.

**Figure 1:**
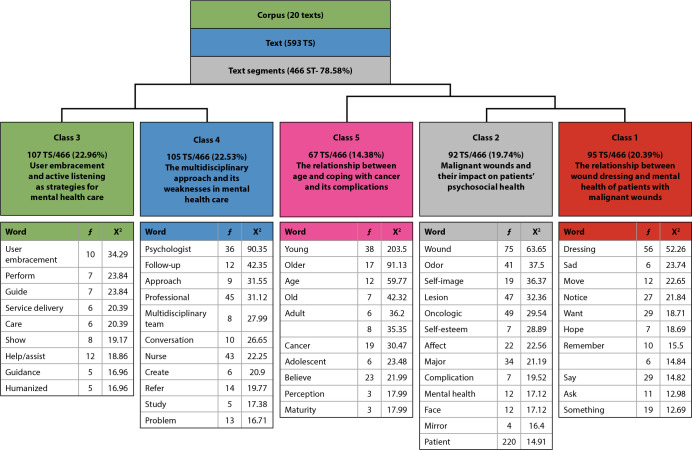
Dendrogram of the Descending Hierarchical Classification of nurses’ perceptions of mental health care for patients with malignant wounds

**Figure 2:**
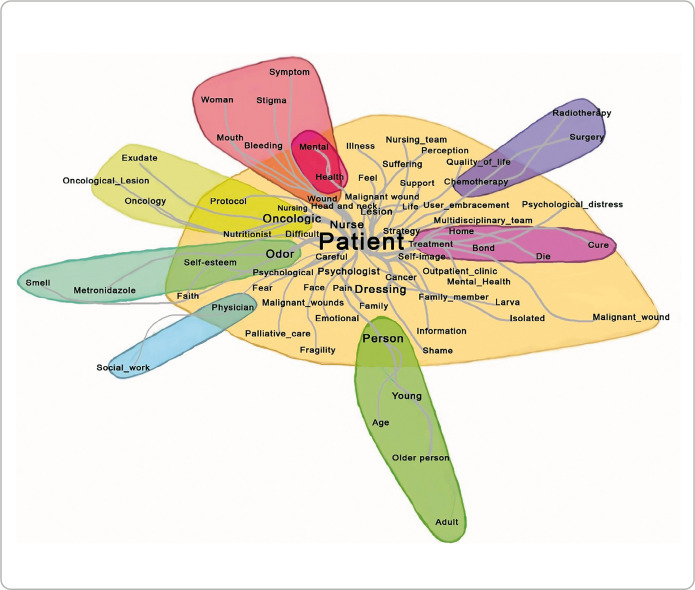
Similarity analysis of nurses’ perceptions of mental health care for patients with malignant wounds

During the exploration stage, we used IRaMuTeQ^(14)^, applying Descending Hierarchical Classification (Figure 1) and a similarity tree (Figure 2). Sociodemographic and professional data from participants were analyzed using the median as the measure of central tendency.

## RESULTS

Twenty nurses who were working or had worked in oncology and had experience providing nursing care to patients with malignant wounds were interviewed. Participant characteristics are presented in Table 1.

**Table 1:** Socioprofessional characteristics of participants, Belém, Pará, Brazil, 2024

Variable	%	n
**Sex** Male Female **Age group** 25-30 31-40 41-50 **Time since graduation (years)** 1-5 5-10 11-20 **Time working in oncology (years)** < 1 1-5 5-10 10-20 ≥ 21 **Practice sector** Public Public and private **Academic credentials** Graduate certificate Master’s Doctorate Postdoctoral TOTAL	8 12 10 7 3 7 8 5 4 9 4 2 1 8 12 11 6 2 1 20	40 60 50 35 15 35 40 25 20 45 20 10 5 40 60 55 30 10 5 100

Nurses’ ages ranged from 25 to 48 years. The median time since graduation was 6.5 years. Time working in oncology ranged from 7 months to 16 years. All participants held a graduate certificate in oncology nursing.

The corpus was then analyzed in IRaMuTeQ, which generated a DHC of 20 texts extracted from the interview transcripts. The corpus was split into 593 text segments; 466 (78.58%) were retained in the DHC.

The most representative words in the dendrogram supported five thematic classes summarizing nurses’ knowledge and perceptions: The relationship between wound dressing and mental health of patients with malignant wounds (Class 1); Malignant wounds and their impact on patients’ psychosocial health (Class 2); User embracement and active listening as strategies for mental health care (Class 3); The multidisciplinary approach and its weaknesses in mental health care (Class 4); and The relationship between age and coping with cancer and its complications (Class 5). Below are selected quotations that illustrate each class.

### The relationship between wound dressing and the mental health of patients with malignant wounds (Class 1)

Nurses recognized the importance of nursing care related to wound dressing. Symptom control and the dressing’s appearance were viewed as key to relieving discomfort, promoting well-being, and, in turn, improving psychological status.

*The dressing’s appearance matters for these patients* […] *a neat, aesthetically pleasing, clean dressing promotes greater comfort; this is confirmed in the literature.* (N4)

*Nursing’s role in performing a high-quality dressing change is essential. It affects the patient’s quality of life and directly their mental health* […] *every dressing must be good, high-quality, and well-finished so patients can look at themselves.* (N11)

Another issue raised concerned the time of the dressing change. Some patients reported it as a period of intense suffering and pain. Strategies are, therefore, needed to make this moment less painful, lighter, and more relaxed, encouraging patients to develop coping mechanisms.

*Whenever he came in for his dressing change, we joked with him* […] *precisely to make that situation as light as possible and to help him see it wouldn’t last forever.* (N2)

*While we were changing the dressing, she chatted with us, joked with us, and said: “Okay, I’m going to sing for you while you change my dressing”. Her mental health was obviously shaken, but she had a way of coping with it.* (N11)

### Malignant wounds and their impact on patients’ psychosocial health (Class 2)

Drawing on nurses’ experiences, malignant wounds worsen mental health, harm self-image, alter self-perception, lower self-esteem, and lead to feelings such as sadness, distress, shame, and inadequacy:

*This patient would only walk around wearing a cap pulled low over his face and a mask because he was embarrassed to show his wounds; whenever anyone spoke to him, his head was down-he was really ashamed.* (N1)

*I think the word that sums up patients with a more advanced malignant wound is fear-fear, anguish, worry. They’re always wearing that worried look.* (N3)

Social relationships were a recurring concern. Symptoms often led patients to avoid contact with others, with malodor standing out as a key driver of isolation for fear of bothering people.

*We couldn’t get the malodor under good control. He felt embarrassed. In his head, for sure, he was thinking: “I must be repulsive to other people”.* (N8)

*He was very ashamed and would ask whether the smell was too strong in the room. Whenever someone came in, he’d say the smell was because he had this wound.* (N10)

Nurses also reported that disfigurement caused by lesions stigmatized patients, who were sometimes rejected or even abandoned because of their appearance and odor:

*He said, “I’m sleeping on the street because my family kicked me out”. I asked if he had tried talking to them to see whether he could go back home; he said, “I don’t want to because her husband threatened me-he said he couldn’t stand me because of my smell and appearance”. So his psychological state was completely shaken, and we were afraid he might attempt to take his own life.* (N2)

### User embracement and active listening: strategies for mental health care (Class 3)

User embracement and active listening were described as core strategies for building rapport with patients and, in turn, for identifying mental health concerns. Nurses emphasized the importance of keeping patients informed about what to expect and dispelling myths surrounding the disease, since clear information strengthens the nurse-patient bond.

*You build rapport and establish a trusting relationship between professional and patient, and from that trust you can start outlining strategies.* (N5)

*It’s a disease full of taboos and fears* […] *because families-and especially patients-don’t get clear information, they often end up without adequate support.* (N8)

Understanding the patient’s socioeconomic context was also seen as essential. Knowing a patient’s life circumstances and level of understanding enables a more tailored approach and effective communication.

*What I notice is that a cancer patient in the public system sees themselves differently from a patient with private insurance* […] *you have to keep that in mind-they’re different groups, and the approach needs to be different.* (N2)

*Know the patient you’re caring for-know their characteristics and pay attention to their lived experience.* (N11)

### The multidisciplinary approach and its weaknesses in mental health care (Class 4)

Nurses recognized how the multidisciplinary team operates and where it falls short in addressing patients’ mental health needs. They emphasized the importance of knowing their scope of practice and promptly engaging other professionals when needed, highlighting the essential role of the psychologist.

*Because nurses have the most frequent, closest contact with patients, they should make timely referrals for psychological support.* (N1)

*I think the nurse’s mistake is listening without acting-listening but not referring the patient to a psychologist.* (N10)

Despite the importance of multidisciplinary collaboration, many challenges persist. Drawing on their experiences, nurses identified barriers such as the limited availability of a multidisciplinary team and gaps in oncology-specific skills.

*At our facility we don’t have an oncology-trained psychologist, so many issues a generalist hears and sees they’ll try to handle the way they’re used to, and that doesn’t always work.* (N2)

*We don’t have a complete multidisciplinary team, so I can’t, for example, rely on a social worker, and these patients have substantial social needs* […] *I don’t have a psychologist either.* (N6)

Interviewees also underscored the nurse’s role in interlinking team members-understanding needs and mediating among professionals as well as between the team and the patient:

*The nurse needs to position themselves as the link within this multidisciplinary team-after all, the nurse is the professional who is with the patient around the clock-so I see nursing as the link that connects this team.* (N11)

### The relationship between age and coping with cancer and its complications (Class 5)

This class presents nurses’ perceptions of how age relates to coping with cancer and its complications. Nurses reported that younger patients tend to experience greater psychological distress, since self-image often carries more weight in youth, and the presence of a malignant wound has a greater impact.

*The impact is greater because of fewer years of life-less experience dealing with situations this difficult-so these wounds may be seen even more negatively.* (N4)

*Younger patients tend to have a harder time* […] *we notice older people don’t usually care as much about looking good* […] *for younger people that matters a lot, so I tend to believe their suffering is a bit greater.* (N5)

Conversely, some nurses emphasized that age itself may not be the decisive factor; instead, the individual’s maturity, family context, and support network are more significant.

*It varies a lot, mainly with our patient’s maturity and the stage of the disease.* (N9)

*Older people are often more susceptible because they may lack support from a family network. We’ve seen many cases where older people were truly abandoned* […] *overall, I think a malignant wound is impactful regardless of age.* (N13)

Figure 2 shows the similarity tree, which displays the relationships among the words “chemotherapy”, “radiotherapy”, and “surgery”. Linked to these words is the term “quality of life”, indicating the importance of implementing and monitoring treatment. In this process, user embracement and clear communication are essential to mitigate treatment-related psychological distress:

*I ran into this patient on the upper floor after he had undergone chemotherapy. The malignant wound wasn’t as large as before; the eye was deformed but flat. I was impressed with how well the chemotherapy worked.* (N8)

*I noticed that after user embracement, the patient went in with less anxiety, knowing what was going to happen. A good user embracement approach, by itself, helps the patient’s mental health.* (N8)

## DISCUSSION

Malignant wounds cause substantial discomfort and are not restricted to biological aspects; their symptoms lead to psychological, emotional, and social deficits^(15)^. In this context, the Theory of Unpleasant Symptoms (TOUS), proposed by Elizabeth Lenz in 1995, helps explain the multidimensional nature of malignant-wound symptoms and shows that this is a complex process with multiple interrelated factors^(16)^.

Psychosocial distress is not always readily perceived by health professionals and may not be outwardly visible, which underscores the need to build a therapeutic relationship between patient and nurse. Studies emphasize that, through in-depth conversations, nurses can identify signs of emotional distress and intervene appropriately^(17)^.

The nursing team also plays a pivotal role in wound management. Evidence highlights the importance of nurses mastering techniques for ongoing care and effective symptom control in patients with malignant wounds^(18)^. In line with TOUS, nurses should understand the different types of symptoms, which supports better planning of interventions^(16)^.

Accordingly, successful control of malodor, exudate, and bleeding is essential to improve patients’ emotional state, since it reduces feelings of shame and, consequently, social isolation^(19)^.

Studies show that malodor in patients with malignant wounds has broad psychosocial repercussions that extend well beyond isolation. It undermines self-esteem and self-confidence, severely restricts daily activities, and is associated with anxiety, fear, and stress. Nurses should therefore implement strategies to mitigate this burden and promote physical, emotional, and social well-being^(20,21)^.

Nurses need to recognize their autonomy in assessing, planning care, and managing symptoms for malignant wounds. In addition to conventional treatments, they may use nonpharmacological strategies for symptom relief. These adjuncts can help alleviate pain and reduce stress, anxiety, and other disorders, while reducing reliance on analgesics and other medications^(22)^.

Considering that all patients with cancer-especially those with malignant wounds-experience mental health harm, age stands out as an aggravating factor. Marink’s study^(23)^ observed that cancer, from diagnosis through complications such as malignant wounds, may have a greater impact on younger patients. Greater emphasis on self-image and social relationships can intensify psychological symptoms, and the sense of losing a life stage may make this process devastating for younger individuals.

A widely discussed issue in mental health nursing care is multidisciplinary care. Studies^(24,25)^ show that care for patients with cancer should not be limited to medical care alone. Comprehensive care requires involving multiple professionals. Despite its importance, this approach faces challenges, such as insufficient training and specialization to address these patients’ specific needs.

The nurse is the central member of the team, acting as the link among professionals and connecting patients and their families with the team. Because nurses provide continuous monitoring, they should identify needs and act to find solutions^(26)^.

### Study limitations

We faced a limitation related to data collection. Some professionals expressed interest but did not feel sufficiently familiar with the mental health topic and declined to participate. Others cited a lack of time to complete the interview. These factors hampered reaching the target number of participants.

### Contributions to the field of nursing

This study offers meaningful contributions for patients and professionals. It encourages reflection on the quality of care delivered and calls attention to the need for comprehensive, humanized care. It also highlights the need for a deeper understanding of the specific needs of patients with cancer and for a care approach tailored to those with malignant wounds.

## FINAL CONSIDERATIONS

Data analysis indicates that, from the nurses’ perspective, mental health care spans multiple aspects-from direct dressing-related care (appearance and symptom control) to understanding intrinsic factors and the patient’s life context.

In their reflections, professionals emphasized nurses’ central role in active listening and user embracement, as well as their role in coordinating members of the multidisciplinary team to deliver comprehensive care. They also underscored the importance of close collaboration with psychologists.

Most interviewees viewed age, socioeconomic context, and support networks as factors influencing patients’ mental health status. They also noted weaknesses related to multidisciplinary provision, such as gaps in team knowledge about the specific needs of patients with cancer, which can affect the quality of care provided.

## Data Availability

The research data are not available.
