# Supplementary material for: Nurses’ perceptions of mental health care for patients with malignant wounds
Source: Rev Bras Enferm. 2026 Jun 12;79:e20250116. doi: 10.1590/0034-7167-2025-0116 (PMC13262984; doi:10.1590/0034-7167-2025-0116)
Supplement: Translation

# Translation: Percepção de enfermeiros sobre a assistência à saúde mental de pacientes com ferida oncológica

**DOI:** 10.1590/0034-7167-2025-0116pt

**Authors:** Daniele Cristina Cordeiro Ferreira da Silva, Aline Mayara Cardoso Baía, Danielle Etienne de Oliveira Bezerra Lima, Dione Seabra de Carvalho, Mary Elizabeth de Santana, Marcos José Risuenho Brito Silva

**Affiliations:** I Faculdade Cosmopolita. Belém, Pará, Brasil; II Universidade do Estado do Pará. Belém, Pará, Brasil

**Keywords:** Enfermagem Oncológica, Saúde Mental, Neoplasias, Ferimentos e Lesões, Cuidados Paliativos.

## Abstract

**Objetivos:**

analisar a percepção de enfermeiros sobre a assistência à saúde mental de pacientes com ferida oncológica.

**Métodos:**

pesquisa descritiva qualitativa; foram entrevistados virtualmente 20 enfermeiros oncológicos mediante Google Meet. Analisaram-se os dados com a análise de conteúdo de Bardin e software IRaMuTeQ (Classificação Hierárquica Descendente e análise de similitude).

**Resultados:**

emergiram cinco classes temáticas: A relação entre curativo e saúde mental; A ferida oncológica e seu impacto na saúde psicossocial dos pacientes; Acolhimento e escuta ativa: estratégias para a assistência à saúde mental; Abordagem multiprofissional e suas fragilidades na assistência à saúde mental; A relação entre idade e enfrentamento do câncer e suas complicações.

**Considerações Finais:**

para os enfermeiros, a assistência à saúde mental permeia aspectos como o curativo, controle dos sintomas e fatores agravantes como a idade e contexto socioeconômico. Destacaram-se a importância da equipe multiprofissional nessa assistência e as fragilidades dessa atuação.

## INTRODUÇÃO

Dentre as complicações associadas ao câncer, encontram-se as feridas oncológicas, que se diferenciam das lesões de outras etiologias por suas particularidades. Em razão do crescimento do tumor e da invasão a tecidos saudáveis, há o comprometimento dos vasos sanguíneos, o que acarreta deficiência na oxigenação, hipóxia e necrose do tecido lesionado. Esse processo favorece a contaminação da área por bactérias e resulta no aumento da quantidade de exsudato e odor fétido^(1)^.

Desde o recebimento do diagnóstico de câncer, a saúde mental do indivíduo sofre grande impacto. Por exemplo, em uma amostra de 100 pacientes, 27% apresentavam prevalência de sintomas depressivos, 24% refletiam indicadores de ansiedade, e 13% tinham indicativos para as duas condições. Desse modo, observa-se que sintomas de depressão e ansiedade são mais prevalentes entre pacientes oncológicos do que no restante da população, por causa da convivência com dores, desconfortos, incertezas e, muitas vezes, deformidades físicas decorrentes de lesões^(2)^.

A ferida oncológica acomete cerca de 5% a 10% dos pacientes em alguma fase da doença^(3)^. Logo, exerce influência direta sobre o estado mental do paciente, que convive com sentimento de angústia provocado pelos sintomas constrangedores característicos da ferida, como odor, pruridos e desfiguração da aparência física, o que gera profundo desconforto e sofrimento. Nessa situação, torna se primordial ao paciente uma assistência adequada para o manejo desses sintomas^(1)^.

A pessoa com ferida oncológica necessita de cuidados diferenciados, considerando a complexidade e especificidade da lesão e os danos causados ao seu bem-estar. Os cuidados a esses pacientes devem ser realizados por uma equipe multiprofissional. Destaca-se a necessidade da assistência de enfermagem no processo de cuidados com a lesão, o que frequentemente se torna uma tarefa complexa para os profissionais, devido às lacunas existentes no conhecimento acerca desse tipo de lesão^(4)^.

Apesar de inúmeras dificuldades, o enfermeiro exerce papel fundamental no cuidado integral ao paciente acometido por ferida oncológica. Por isso, a equipe de enfermagem permanece ao lado do indivíduo em tempo integral, favorecendo maior proximidade e entendimento de suas necessidades. Além das etapas inerentes ao Processo de Enfermagem, o enfermeiro pode utilizar a escuta ativa como uma ferramenta para auxiliar o doente em suas demandas biopsicossociais^(5)^.

A produção científica sobre a temática foi identificada por meio de uma busca na base de dados PubMed/MEDLINE, utilizando os descritores “*Palliative Care*”, “*Injury*”, “*Wound*”, “*Neoplasms*”, “*Cancer*”, “*Mental Health*” e “*Nursing*” associados aos operadores booleanos “AND” e “OR”, sem recorte temporal. A busca retornou oito publicações. Após análise de títulos e resumos, identificou-se apenas um estudo relacionado ao declínio da qualidade de vida e da saúde mental de pacientes com feridas oncológicas, porém tal trabalho foi realizado na perspectiva de pacientes^(6)^. Esse resultado evidencia a necessidade de estudos que abordem a assistência de enfermagem direcionada especificamente à saúde mental de pacientes com ferida oncológica.

Diante disso, analisar a percepção de enfermeiros acerca do assunto é essencial para produzir evidências que contribuam para uma melhor assistência aos pacientes com ferida oncológica.

## OBJETIVOS

Analisar a percepção de enfermeiros sobre a assistência à saúde mental de pacientes com ferida oncológica.

## MÉTODOS

### Aspectos éticos

A pesquisa observou as normas dispostas na Resolução nº 510, de 7 de abril de 2016^(7)^, e os princípios éticos descritos na Resolução nº 466, de 12 de dezembro de 2012, ambas do Conselho Nacional de Saúde^(8)^. O projeto foi aprovado pelo Comitê de Ética em Pesquisa (CEP). O Termo de Consentimento Livre e Esclarecido (TCLE), assim como o Termo de Autorização para Gravação de Voz, foram preenchidos e assinados por todos os participantes de forma eletrônica.

### Tipo de estudo

Trata-se de uma pesquisa descritiva com abordagem qualitativa. A pesquisa descritiva é um método que consiste na descrição do fenômeno ocorrido em um grupo ou população, enquanto a abordagem qualitativa é focada na compreensão de ideias subjetivas. Esse tipo de estudo parte da premissa de que o conhecimento humano só pode ser compreendido por meio das experiências humanas descritas por seus próprios protagonistas^(9,10)^. Em todas as fases da pesquisa, forma considerados os critérios estabelecidos no *Consolidated Criteria for Reporting Qualitative Research* (COREQ)^(11)^.

### Cenário de estudo

O estudo foi realizado em ambiente virtual por meio da versão gratuita da plataforma *Google Meet*, o que possibilitou facilidade no acesso, maior alcance geográfico dos profissionais e maior conforto, pois participaram de sua residência no momento mais oportuno. A pesquisa ocorreu sem a necessidade de vínculo institucional dos participantes.

### Fonte de dados

Os participantes foram contatados por meio do aplicativo de mensagens WhatsApp, no qual receberam o convite para participar da pesquisa. No contato inicial, a equipe apresentou o projeto, explicou os objetivos e a justificativa do estudo e detalhou a metodologia, favorecendo a criação de vínculo prévio entre os profissionais e os pesquisadores.

A técnica bola de neve^(10)^ foi usada para compor a amostra, possibilitando o acesso aos contatos dos participantes por intermédio de referências e indicações. Foram incluídos enfermeiros oncológicos e residentes de oncologia que atuavam ou haviam atuado na assistência a pacientes com ferida neoplásica por um período maior que três meses. Foram excluídos os enfermeiros que estavam de férias ou licença-saúde durante a coleta. Dentre os 56 convidados, 32 recusaram-se a participar e 4 desistiram antes da entrevista, alegando falta de disponibilidade.

A amostra foi definida por saturação, ou seja, a coleta de dados era finalizada quando os discursos não acrescentavam novas informações relevantes^(12)^. Desse modo, a amostra foi de 20 enfermeiros.

### Coleta e organização dos dados

A coleta de dados ocorreu nos meses de outubro e novembro de 2024, por meio de entrevistas virtuais individuais conduzidas por duas graduandas de Enfermagem. Cada entrevista teve duração média de 20 minutos e contou com gravação para posterior transcrição. Todos os dados foram arquivados em nuvem e serão mantidos por um período de cinco anos, após o qual serão deletados. O anonimato dos participantes foi garantido utilizando a letra “E” (de “enfermeiro”) seguida de um número arábico (E1, E2, E3…).

Para a coleta, aplicou-se um roteiro de entrevista semiestruturado contendo 13 perguntas que tratavam da formação e atuação profissional, experiência no setor público ou privado e faixa etária. Havia também questões sobre os abalos psicológicos observados em seus pacientes e seus possíveis fatores desencadeantes, estratégias utilizadas para minimizar tais danos, desafios relacionados à atuação na equipe multiprofissional, entre outras, que possibilitaram ao participante explanar livremente suas percepções e experiências.

### Análise dos dados

A transcrição ocorreu com o uso da função “digitação por voz” do processador de texto *on line Google Docs*, que transcreveu literalmente as falas. Em seguida, todos os dados foram agrupados destacando os principais pontos ligados ao tema. As entrevistas foram analisadas e categorizadas com a utilização da análise de conteúdo proposta por Laurence Bardin, que estabelece três etapas: pré análise; exploração do material; e tratamento dos dados e interpretação^(13)^.

Na etapa de exploração do material, utilizou-se o *software Interface de R pour les Analyses Multidimensionnelles de Textes et de Questionnaires* (IRaMuTeQ)^(14)^, com emprego da Classificação Hierárquica Descendente (CHD) de palavras (Figura 1) e árvore de similitude (Figura 2). Quanto aos dados socioprofissionais dos entrevistados, foram submetidos à análise por meio da mediana como medida de tendência central.

**Figura 1:** Dendrograma da Classificação Hierárquica Descendente referente à percepção dos enfermeiros sobre a assistência à saúde mental de pacientes com feridas oncológicas

**Figura 2:** Análise de similitude referente à percepção dos enfermeiros sobre a assistência à saúde mental de pacientes com feridas oncológicas

## RESULTADOS

Foram entrevistados 20 enfermeiros que atuavam ou haviam atuado na área da oncologia, com experiência na assistência de enfermagem a pacientes com ferida oncológica. A caracterização dos participantes é apresentada na Tabela 1.

**Tabela 1:** Características socioprofissionais dos participantes, Belém, Pará, Brasil, 2024

Variável	%	n
**Sexo** Masculino Feminino **Faixa etária** 25-30 31-40 41-50 **Tempo desde a formação (anos)** 1-5 5-10 11-20 **Tempo de atuação na oncologia (anos)** < 1 1-5 5-10 10-20 ≥ 21 **Setor de atuação** Público Público e privado **Títulos** Especialistas Mestres Doutores Pós-doutores TOTAL	8 12 10 7 3 7 8 5 4 9 4 2 1 8 12 11 6 2 1 20	40 60 50 35 15 35 40 25 20 45 20 10 5 40 60 55 30 10 5 100

A idade dos enfermeiros variou de 25 a 48 anos. A mediana do tempo de formação foi de 6,5 anos. Quanto à atuação na oncologia, o tempo variou entre 7 meses e 16 anos. Todos os participantes tinham especialização em Enfermagem oncológica.

Posteriormente, foi realizada a análise do corpus pelo software IRaMuTeQ, no qual foi gerada uma CHD de 20 textos extraídos da transcrição das entrevistas. Para isso, o corpus foi separado em 593 segmentos de texto (ST); na CHD, foram aproveitados 466 STs (78,58%).

As palavras de maior representação no dendrograma permitiram elaborar cinco classes temáticas apresentando os conhecimentos e percepções dos enfermeiros: A relação entre curativo e saúde mental do paciente com ferida oncológica (Classe 1), A ferida oncológica e seu impacto na saúde psicossocial dos pacientes (Classe 2), Acolhimento e escuta ativa: estratégias para a assistência à saúde mental (Classe 3), Abordagem multiprofissional e suas fragilidades na assistência à saúde mental (Classe 4) e A relação entre idade e enfrentamento do câncer e suas complicações (Classe 5). A seguir, são apresentadas algumas falas dos entrevistados que ilustram e embasam as respectivas classes.

### A relação entre curativo e saúde mental do paciente com ferida oncológica (Classe 1)

Os enfermeiros reconheceram a importância dos cuidados de enfermagem relacionados ao curativo. O controle dos sintomas e a boa estética do curativo mostram se fatores fundamentais para o alívio do incômodo, promovem o bem-estar aos pacientes e, por consequência, melhoram seu estado psicológico.

*A estética do curativo é importante para esse paciente* […] *então o curativo bonito, estético, limpo promove mais conforto; isso é confirmado na literatura.* (E4)

*A influência da enfermagem na realização de um curativo bem feito é essencial. Isso vai interferir na qualidade de vida do paciente e diretamente na saúde mental* […] *todo curativo precisa ser bom, ter qualidade, ser bonito e bem feito em seu acabamento, para que ele* [paciente] *consiga se olhar.* (E11)

Outro ponto observado pelos entrevistados relaciona-se com o momento da realização do curativo. Alguns pacientes afirmaram que são instantes de grande sofrimento e dor. Por isso, são necessárias estratégias para que esse momento seja menos doloroso, mais leve e mais descontraído, criando e incentivando o paciente a criar mecanismos de enfrentamento da situação.

*Sempre que ele entrava para fazer a curativo, a gente brincava com ele* […] *que era justamente para tornar aquela condição a mais leve possível e para que ele visse que aquilo não iria ser para sempre.* (E2)

*No momento que a gente fazia a troca do curativo, ela conversava com a gente, brincava com a gente, ela falava: “Ah, então eu vou cantar para vocês enquanto vocês fazem o meu curativo”. Obviamente a saúde mental dela estava abalada, mas ela tinha um manejo para aquilo.* (E11)

### A ferida oncológica e seu impacto na saúde psicossocial dos pacientes (Classe 2)

De acordo com as experiências dos enfermeiros, a ferida neoplásica é um fator agravante na saúde mental, compromete a autoimagem, modifica a autopercepção, diminui a autoestima e leva a sentimentos como tristeza, angústia, vergonha e inadequação. Alguns participantes confirmam isso com os relatos a seguir:

*Esse paciente só andava com boné cobrindo o rosto e com máscara, porque ele tinha vergonha de mostrar as feridas dele; e todo tempo que se falava com ele era com a cabeça abaixada, porque ele tinha muita vergonha disso.* (E1)

*Acho que a palavra que envolve os pacientes que tem uma ferida oncológica num estágio mais avançado é justamente o medo, a angústia, a preocupação. Eles ‘tão sempre com essa expressão assim de preocupação.* (E3)

Um importante fator mencionado são os relacionamentos sociais. Por vezes, os sintomas fazem com que o paciente evite contato com outras pessoas. Destaca-se o odor como um dos principais sintomas que levam ao isolamento pelo medo de importunar outras pessoas.

*A gente não conseguia um bom controle do odor. Ele se sentia envergonhado. Com certeza, dizia na cabeça dele: “Eu devo estar causando repulsa nas outras pessoas”.* (E8)

*Ele tinha muita vergonha e ele perguntava se o fedor estava muito forte no quarto. Toda pessoa que chegava, ele dizia que o cheiro era por conta que ele tinha essa ferida.* (E10)

Os enfermeiros afirmam que a desfiguração causada pelas lesões coloca sobre o paciente um grande estigma: são muitas vezes desprezados e até mesmo abandonados por causa da aparência e odor. Tal situação é descrita no relato a seguir:

*Ele falou: “Estou dormindo na rua porque a minha família me expulsou de casa”. Eu perguntei se ele já tinha ido conversar para saber se ele podia voltar para casa; ele disse: “Eu não quero fazer isso porque o marido dela me ameaçou, disse que não gostava de mim por conta do meu cheiro e aparência”. Então, o psicológico dele estava completamente comprometido, e a gente ficava com medo dele atentar contra a própria vida.* (E2)

### Acolhimento e escuta ativa: estratégias para a assistência à saúde mental (Classe 3)

O acolhimento e a escuta ativa foram abordados como estratégias primordiais para estabelecer um vínculo com o paciente e, com isso, detectar problemas relacionados à saúde mental. Nesse sentido, os enfermeiros ressaltaram a importância de mantê-lo esclarecido acerca do que lhe ocorrerá e desfazer os mitos que circundam a doença, já que esse esclarecimento colabora para a construção do vínculo entre profissional e paciente.

*Você cria vínculo e estabelece uma relação de confiança entre profissional e paciente e, a partir desse estabelecimento de vínculo de confiança, a gente consegue começar a traçar estratégias.* (E5)

*É uma doença que é muito cheia de tabus, medos* […] *por falta de informações bem dadas para essa família e principalmente para o paciente, eles ficam muito desassistidos.* (E8)

A compreensão do contexto socioeconômico do paciente torna-se importante nesse processo. Segundo os enfermeiros, compreender seu contexto de vida e seu nível de entendimento facilita o alcance de uma melhor abordagem e comunicação eficaz.

*O que eu percebo é que o paciente oncológico do SUS se enxerga de uma forma diferente daquele paciente que tem um convênio* […] *tem que ter isso em mente, que são públicos diferentes, e a abordagem tem que ser diferente.* (E2)

*Conhecer o paciente o qual a gente está atendendo, conhecer as características desse paciente, observar o seu aspecto enquanto vivência.* (E11)

### Abordagem multiprofissional e suas fragilidades na assistência à saúde mental (Classe 4)

Os enfermeiros perceberam como atua equipe multiprofissional e suas fragilidades na atenção à saúde mental dos pacientes. Destacou-se a importância de o enfermeiro estar consciente de suas competências em relação à temática e acionar outros profissionais sempre que necessário, destacando o papel imprescindível do psicólogo.

*O enfermeiro, como ele tem esse contato mais frequente, mais próximo com o paciente, ele deve procurar encaminhar de forma mais rápida possível à ajuda psicológica.* (E1)

*Eu acho que o erro do enfermeiro é ouvir sem atuar: você ouvir, mas sem encaminhar o paciente para um psicólogo.* (E10)

Apesar da grande importância da atuação multiprofissional, muitos desafios são constantemente enfrentados. As experiências dos enfermeiros permitiram que eles apontassem enquanto entraves a falta de disponibilidade de uma equipe multiprofissional eficientemente implementada e a ausência de algumas habilidades específicas para pacientes oncológicos.

*No nosso serviço, nós não temos psicólogo com especialidade em oncologia, então muitas coisas que o psicólogo generalista vai escutar e vai ver, ele vai tentar resolver da forma que ele está habituado, e nem sempre isso vai dar certo.* (E2)

*É porque você não dispõe de uma equipe multiprofissional completa, então eu não consigo, por exemplo, ter na minha disposição uma assistente social, e essas pessoas têm demandas sociais muito grandes* […] *eu não consigo ter uma psicóloga.* (E6)

Os entrevistados ressaltaram a importância de o enfermeiro entender o seu papel de interligação dos membros da equipe, compreendendo as necessidades e fazendo a mediação entre o paciente e os demais profissionais:

*O enfermeiro precisa se colocar como elo dessa equipe multiprofissional, até porque é o enfermeiro o profissional que acompanha 24 horas por dia a vida do paciente, então eu vejo o profissional de enfermagem como o elo conectando essa equipe.* (E11)

### A relação entre idade e enfrentamento do câncer e suas complicações (Classe 5)

A presente classe descreve a percepção dos enfermeiros com relação à faixa etária e ao enfrentamento da doença e seus agravos. Foi relatado que o público mais jovem tende a sofrer um maior abalo psicológico, pois entende-se que a autoimagem seja mais valorizada na juventude e a presença da ferida oncológica tenha maior impacto.

*O impacto é maior por conta dos anos vividos - a menor experiência que ele tem para lidar com situações tão difíceis - então essas lesões podem ser encaradas de forma mais negativa ainda.* (E4)

*Os pacientes mais jovens tendem a sofrer um pouco mais* […] *a gente nota que idosos não costumam ligar mais tanto pra estarem bonitos* […] *para os jovens isso significa muito, então eu tendo a acreditar que, para os jovens, esse sofrimento é um pouco maior.* (E5)

Em contrapartida, alguns enfermeiros salientam que a faixa etária pode não ser um fator de maior influência, e sim a maturidade do indivíduo, seu contexto familiar e rede de apoio.

*Isso varia muito, principalmente da maturidade do nosso paciente e do estadiamento que esteja a doença.* (E9)

*Muitas vezes, o idoso é mais suscetível, pela falta de apoio de uma rede familiar. A gente já viu muitos casos de idosos que eram realmente abandonados* […] *eu acho, de uma forma geral, que a ferida oncológica é impactante independente da faixa etária.* (E13)

Na Figura 2, observa-se a árvore de similitude, em que é demonstrada uma relação entre as palavras “quimioterapia”, “radioterapia” e “cirurgia”. Interligado a essas palavras, encontra-se o termo “qualidade de vida”, indicando também a importância de implementar e acompanhar os tratamentos. Torna-se importante, nesse processo, utilizar o acolhimento e a comunicação para amenizar o sofrimento psicológico causado pelo tratamento. Tal análise é exemplificada nos relatos a seguir:

*Eu encontrei esse paciente no andar superior, depois de ter feito quimioterapia. A ferida tumoral não estava mais do tamanho que estava, o olho estava deformado, mas plano. Eu fiquei impressionada pelo bom resultado que deu a quimioterapia.* (E8)

*Eu percebi que, depois do acolhimento, o paciente já ia com menos ansiedade, sabendo o que ia acontecer. A estratégia de fazer um bom acolhimento já vai ajudar na saúde mental do paciente.* (E8)

## DISCUSSÃO

A ferida oncológica gera grande desconforto e não se restringe aos aspectos biológicos: seus sintomas acarretam déficits psicológico, emocional e social^(15)^. Nesse contexto, com base na Teoria dos Sintomas Desagradáveis (TOUS), proposta por Elizabeth Lens em 1995, pode-se compreender a multidimensionalidade dos sintomas da ferida oncológica e vislumbrar que é um processo de acometimento complexo com vários fatores inter-relacionados^(16)^.

Por vezes não facilmente percebida pelos profissionais de saúde, a dor psicossocial pode não se manifestar de maneira visível, o que torna necessária a criação de uma relação entre o paciente e o enfermeiro. Estudos ressaltam que, por meio de conversas mais profundas, o enfermeiro conseguirá identificar sinais de dor emocional e agir adequadamente^(17)^.

Além disso, a equipe de enfermagem é peça fundamental no manejo das lesões. Estudo destacou a importância de o enfermeiro dominar as técnicas de manutenção e controle efetivo dos sintomas da ferida oncológica^(18)^. De acordo com a Teoria dos Sintomas Desagradáveis, o enfermeiro precisa ter conhecimento dos diversos tipos de sintomas, o que contribui para um melhor planejamento de suas intervenções^(16)^.

Diante disso, vale ressaltar que o êxito no controle do odor, exsudato e sangramento é essencial para melhorar o aspecto emocional do paciente, pois diminui o sentimento de vergonha e, consequentemente, reduz o isolamento social^(19)^.

Estudos realizados mostram que o odor em pacientes com ferida oncológica tem ampla repercussão psicossocial: seus impactos vão muito além do isolamento. O odor gera baixa autoestima, perda da autoconfiança, graves limitações nas atividades cotidianas, além de ansiedade, medo e estresse. Por essa razão, o enfermeiro deve lançar mão de estratégias para melhorar esse quadro, visando ao bem-estar físico, emocional e social^(20,21)^.

É importante que o enfermeiro compreenda sua autonomia na avaliação, prescrição do cuidado e controle dos sintomas da ferida neoplásica. Além dos tratamentos convencionais, ele pode utilizar estratégias não farmacológicas para alívio de sintomas. Esses recursos podem ser usados de forma complementar para o alívio da dor e redução do estresse, ansiedade e outros transtornos, além de diminuir o impacto causado por analgésicos e outras medicações^(22)^.

Ao considerar que todos os pacientes oncológicos, sobretudo os que têm uma ferida oncológica, sofrem danos à saúde mental, destaca-se a faixa etária como um fator que agrava o sofrimento. Estudo de Marink^(23)^ observou que o câncer, desde seu diagnóstico até as suas complicações como a ferida oncológica, pode ter um impacto maior em pacientes mais jovens. A maior valorização da autoimagem e das relações sociais agrava os sintomas psicológicos, e o sentimento de perda de uma fase da vida pode tornar esse processo devastador para o paciente jovem.

Uma questão amplamente debatida com relação à assistência de enfermagem à saúde mental é a atenção multiprofissional. Estudos^(24,25)^ mostram que o cuidado prestado ao paciente oncológico não deve se limitar ao saber médico. Essa assistência precisa ser prestada de maneira integral; para isso, é necessário envolver diversos profissionais no processo. Apesar de fundamental, essa assistência enfrenta desafios, como a falta de capacitação e especialização para o manejo das demandas especificas desses pacientes.

O enfermeiro é a peça central da equipe, atuando como um elo entre os profissionais e conectando o paciente e seus familiares com a equipe. Por exercer a vigilância contínua do paciente, ele deve observar as demandas e atuar com a finalidade de encontrar a solução^(26)^.

### Limitações do estudo

Houve uma limitação relacionada à coleta de dados. Alguns profissionais demostraram interesse, porém não se sentiram familiarizados com a temática da saúde mental, o que resultou na recusa em participar. Outros, por sua vez, alegaram falta de tempo para a realizar a entrevista. Tais situações refletiram-se na dificuldade em alcançar o número desejado de participantes.

### Contribuições para Área da Enfermagem

O presente estudo fornece contribuições significativas para pacientes e profissionais. Gera reflexões acerca da qualidade da assistência ofertada e desperta um olhar para a necessidade de um cuidado humanizado e integral. Além disso, evidencia a necessidade de maior aprofundamento a respeito das particularidades do paciente oncológico e de uma abordagem de cuidados específica para aquele com ferida oncológica.

## CONSIDERAÇÕES FINAIS

A análise dos dados mostrou que a assistência à saúde mental na perspectiva dos enfermeiros permeia diversos aspectos. Eles vão desde os cuidados diretos relacionados ao curativo, como boa estética e controle adequado dos sintomas, até a importância de compreender fatores intrínsecos e o contexto de vida do paciente.

Em suas reflexões, os profissionais mencionaram a função do enfermeiro como peça fundamental na escuta e acolhimento dos pacientes, bem como seu papel na articulação entre os membros da equipe multiprofissional para promover assistência integral. Destacaram ainda a importância do trabalho conjunto com o psicólogo.

A maioria dos entrevistados considerou a faixa etária, o contexto socioeconômico e a rede de apoio como fatores influenciadores do quadro mental dos pacientes. Além disso, foram mencionadas as fragilidades relacionadas à equipe multiprofissional, como a falta de conhecimento da equipe sobre as demandas específicas do paciente oncológico, o que afeta a qualidade da assistência oferecida.

## Data Availability

Os dados de pesquisa não estão disponíveis.
